# Supplementary material for: The media risk of infodemic in public health emergencies: Consequences and mitigation approaches
Source: PLoS One. 2024 Sep 12;19(9):e0308080. doi: 10.1371/journal.pone.0308080 (PMC11392340; doi:10.1371/journal.pone.0308080)
Supplement: S1 Dataset — (PDF) [file pone.0308080.s001.pdf]

	Information Attention					Opinion Leaders			Risk Perception				Information Sharing Willingness			Perceived Usefulness		
ID	IA1	IA2	IA3	IA4	IA5	OL1	OL2	OL3	RP1	RP2	RP3	RP4	ISW1	ISW2	ISW3	PU1	PU2	PU3
1	5	4	4	4	3	4	3	4	5	4	5	4	5	4	5	4	5	5
2	4	4	3	4	4	4	4	3	5	5	5	5	4	4	5	4	4	4
3	4	4	3	5	4	3	2	3	4	2	4	3	4	4	4	4	3	4
4	4	4	5	4	4	4	4	4	5	5	5	5	4	4	4	5	5	4
5	3	4	4	4	4	3	3	3	5	5	5	4	3	3	3	3	4	3
6	4	4	3	5	4	3	3	3	4	3	4	3	3	3	3	3	3	3
7	4	4	4	4	4	4	5	5	5	4	4	4	3	3	3	3	4	4
8	5	4	4	5	4	4	3	4	5	5	5	5	4	3	4	4	3	3
9	3	3	3	3	2	3	4	3	5	4	4	4	4	3	3	4	4	4
10	5	4	5	5	3	5	5	5	5	5	5	5	5	5	4	5	4	5
11	5	5	5	5	5	5	5	3	4	2	3	3	3	5	3	3	3	4
12	3	3	3	3	3	3	3	3	4	3	4	3	3	3	3	3	3	3
13	5	5	5	5	5	2	2	2	4	4	4	4	4	4	4	3	3	3
14	5	5	5	5	4	3	3	4	4	3	3	2	5	5	4	3	3	3
15	4	4	3	4	4	3	3	3	5	4	5	3	4	5	4	3	3	3
16	5	5	5	5	5	5	4	4	5	3	4	3	5	5	5	4	4	5
17	1	1	1	1	1	1	1	1	1	1	1	1	1	1	1	1	1	1
18	5	5	5	5	5	5	4	5	5	4	5	5	5	5	5	5	5	5
19	4	4	4	4	3	3	3	2	5	4	5	3	4	3	3	3	3	3
20	5	5	5	5	5	4	4	4	5	5	5	4	5	5	3	4	4	4
21	4	4	4	4	4	3	3	3	4	4	4	4	4	4	4	3	3	3
22	4	4	5	5	4	3	3	3	5	5	5	3	4	4	4	3	4	4
23	3	3	3	3	3	3	3	3	4	4	4	5	1	4	2	4	3	2
24	4	5	5	5	5	3	3	3	5	5	5	4	5	5	5	3	3	3
25	4	4	5	5	4	3	3	4	5	5	5	4	5	4	5	4	4	4
26	5	4	5	4	4	2	2	3	4	4	4	3	3	3	4	1	2	2
27	3	3	3	3	3	3	3	3	5	5	5	5	3	3	3	5	5	5
28	4	4	4	4	4	4	4	4	4	4	4	4	4	4	4	4	4	4
29	5	5	5	5	5	5	5	4	5	5	5	5	5	5	5	4	4	5
30	4	5	5	5	5	5	3	3	4	4	4	2	4	5	4	3	4	4
31	5	5	5	4	5	5	5	5	5	5	5	5	5	5	5	3	3	4
32	5	5	4	5	5	3	3	3	5	3	4	2	5	5	4	3	3	3
33	5	5	4	5	3	3	3	3	5	5	5	5	3	4	3	5	5	5
34	4	5	4	5	5	5	4	5	5	4	5	5	4	5	5	5	5	5
35	4	4	4	4	4	4	4	3	4	4	4	4	5	4	4	4	4	4
36	4	4	3	4	5	5	3	4	5	2	4	3	4	4	5	4	5	4
37	4	4	4	4	4	4	4	3	4	3	4	3	3	4	3	3	4	4
38	4	4	4	4	3	4	4	4	5	3	4	3	4	4	4	4	4	4
39	4	4	3	5	4	4	4	4	4	3	2	3	3	4	4	3	3	4
40	4	4	4	4	4	4	4	4	5	4	5	3	4	4	4	3	4	4
41	5	5	5	5	5	5	5	5	5	5	5	5	5	5	5	5	5	5
42	5	5	5	5	3	5	4	4	5	3	4	4	4	4	4	5	4	5
43	5	5	5	5	5	4	3	3	5	5	5	4	5	5	5	3	4	4
44	4	4	4	5	5	3	3	3	4	2	4	2	4	4	4	4	4	4
45	5	4	4	5	3	4	5	3	5	4	5	5	5	5	5	4	4	4
46	5	4	4	5	4	4	3	4	4	4	5	4	4	4	5	4	4	4
47	5	5	4	5	5	5	5	5	5	5	5	5	5	5	5	5	5	5
48	3	3	3	3	3	3	3	3	3	3	3	3	3	3	3	3	3	3
49	4	4	3	4	2	5	5	5	4	4	4	4	4	4	4	2	3	3
50	4	3	4	4	4	3	3	3	4	3	4	3	3	3	3	3	3	3
51	5	5	5	5	5	3	3	3	5	5	5	5	5	5	5	3	4	3
52	5	5	5	5	5	3	3	2	4	3	4	2	4	4	4	3	3	4
53	3	3	3	3	3	3	3	3	3	3	3	3	3	3	3	3	3	3
54	5	3	5	4	4	3	3	3	5	4	5	3	3	3	3	3	4	4
55	4	4	4	4	4	5	4	3	4	4	4	4	4	4	4	4	4	4
56	5	4	4	4	5	5	5	5	4	5	5	5	4	5	5	5	5	4
57	5	4	4	4	5	4	4	3	4	3	4	3	4	4	4	3	4	4
58	5	5	5	5	5	4	3	4	5	4	4	4	5	5	4	4	4	4
59	5	5	4	4	4	5	3	4	5	4	4	4	5	5	5	4	4	4
60	4	4	4	4	2	5	5	5	5	3	4	2	5	5	4	3	4	5
61	5	5	4	4	4	5	5	5	4	4	5	4	5	4	4	4	4	2
62	3	4	4	4	3	4	4	4	5	5	4	4	3	4	4	4	4	3
63	3	4	4	4	4	4	4	4	5	4	5	4	4	5	5	4	4	4
64	5	5	5	5	5	5	5	5	5	5	5	5	5	5	5	5	5	5
65	4	4	5	4	4	5	5	5	5	4	5	4	4	2	4	5	5	5
66	4	4	4	3	3	3	3	3	5	4	4	3	3	3	3	3	3	3
67	4	4	4	4	4	4	4	4	3	2	3	2	4	4	4	4	4	4
68	4	4	4	4	4	4	3	4	5	5	5	4	5	5	4	4	4	4
69	5	5	5	5	4	3	3	4	4	3	4	2	4	5	4	4	5	4
70	4	4	3	5	4	4	3	4	4	4	5	3	4	4	2	4	4	4
71	5	5	5	5	5	5	5	5	5	5	5	5	5	5	5	5	5	5
72	3	4	4	4	4	4	4	4	4	4	4	4	4	4	4	3	3	3
73	4	4	4	4	4	4	4	4	5	4	5	4	4	4	4	4	4	4
74	4	4	3	4	4	4	4	4	5	5	5	5	5	5	5	4	4	4
75	4	4	3	4	4	5	5	5	5	5	5	5	4	4	4	4	4	4
76	4	4	4	4	3	5	5	5	4	3	4	3	3	4	3	3	3	4
77	5	5	5	5	5	5	5	5	5	5	5	5	5	5	5	5	5	5
78	4	4	4	4	4	3	3	3	4	3	4	3	3	3	3	3	3	3
79	4	4	4	4	4	4	4	4	4	4	4	4	4	4	4	4	4	4
80	5	5	5	5	5	4	3	5	4	3	4	2	4	3	4	3	4	4
81	4	4	4	4	4	4	3	4	4	4	4	4	4	4	3	4	4	4
82	5	5	5	5	5	3	3	4	4	2	4	2	5	5	4	3	3	3
83	4	4	5	5	5	3	4	4	5	5	5	5	5	5	5	1	1	1
84	4	4	4	4	4	3	3	4	5	3	3	4	4	3	4	3	3	3
85	4	4	4	4	4	3	3	3	4	2	4	4	3	3	3	3	3	3
86	4	4	4	5	5	3	3	3	4	3	4	4	4	3	2	3	4	4
87	1	1	2	2	1	2	4	2	3	4	4	4	4	4	5	3	2	3
88	5	5	5	5	5	5	5	5	5	5	5	5	5	5	5	5	5	5
89	5	4	3	4	3	3	3	3	4	2	3	2	3	3	3	3	3	3
90	5	5	5	5	5	5	5	5	5	5	5	5	5	5	5	5	5	5
91	5	5	5	5	5	3	4	4	5	5	5	3	3	4	4	4	4	4
92	5	5	5	5	5	3	3	2	5	5	5	4	4	4	3	2	3	3
93	4	4	4	5	5	3	3	3	5	4	4	4	4	5	4	3	4	4
94	5	3	5	5	5	4	4	4	5	4	5	4	5	5	3	4	4	5
95	5	4	5	5	5	4	4	4	5	2	5	2	5	5	5	4	4	5
96	3	3	4	4	4	4	4	3	4	3	4	2	4	4	4	3	4	4
97	4	4	4	4	4	4	4	4	4	4	4	4	4	4	4	4	4	4
98	5	5	5	4	5	2	3	1	2	2	3	1	3	4	4	3	3	3
99	5	5	5	5	5	4	4	4	4	4	4	5	4	4	4	4	4	4
100	1	1	4	5	5	4	4	4	5	4	5	4	4	5	4	4	4	4
101	4	4	4	4	3	3	3	3	4	4	4	2	3	3	4	3	3	3
102	5	5	5	5	5	3	3	3	5	3	5	2	5	5	3	3	3	3
103	4	4	4	4	3	3	5	5	4	5	5	4	4	4	5	3	4	4
104	4	3	2	2	2	3	3	3	4	3	3	3	4	2	3	3	4	4
105	5	4	5	5	4	4	3	4	5	3	5	3	5	5	5	4	5	5
106	5	5	5	5	5	5	5	5	5	4	4	4	4	5	5	5	5	5
107	5	5	5	5	5	4	4	4	5	4	4	4	4	4	4	4	4	4
108	1	1	1	1	1	5	5	5	1	1	1	1	1	1	1	3	3	2
109	5	5	5	5	5	4	3	3	4	5	5	3	3	3	3	3	3	3
110	5	4	5	4	4	4	3	3	3	4	3	3	4	4	3	3	4	4
111	4	4	4	4	4	4	4	4	4	4	4	4	4	4	4	4	4	4
112	5	5	5	5	5	5	5	5	4	5	5	5	5	5	5	5	5	5
113	5	5	5	5	5	4	4	5	5	3	5	2	5	5	4	4	4	4
114	5	5	5	5	5	5	5	5	5	5	5	5	5	5	5	5	5	5
115	4	5	4	4	4	2	3	3	4	4	4	3	4	4	3	3	4	4
116	3	4	4	3	3	3	3	1	5	3	4	3	4	4	4	3	3	3
117	4	4	4	4	4	2	3	3	4	4	4	3	4	4	4	3	4	3
118	4	4	3	4	2	1	1	1	1	1	1	1	2	2	2	2	2	2
119	1	1	1	1	1	3	3	2	2	1	3	1	2	2	2	1	1	1
120	5	4	3	5	5	3	4	4	3	3	4	3	4	4	3	4	3	3
121	5	5	5	5	5	4	4	4	4	4	4	4	5	5	4	4	4	4
122	4	4	4	4	4	4	4	4	4	4	4	4	4	4	3	4	4	4

123	4	4	4	4	4	4	4	4	4	3	4	3	4	4	4	4	4	4
124	5	5	5	5	5	5	5	5	5	5	5	2	5	5	5	5	4	4
125	5	3	5	5	5	5	5	5	5	5	5	5	5	5	3	5	5	5
126	5	5	5	5	5	5	3	3	5	5	5	3	5	5	5	3	3	3
127	5	5	5	5	5	5	5	5	5	5	5	3	5	5	5	3	5	5
128	4	4	3	4	2	4	2	4	5	4	4	4	4	3	4	3	4	3
129	5	5	4	5	3	5	3	5	3	4	4	3	5	5	5	5	5	5
130	4	4	4	4	4	4	4	3	4	4	4	4	4	3	5	3	4	4
131	4	4	4	4	4	4	4	3	5	5	5	4	4	5	4	4	4	4
132	3	3	3	4	4	4	3	3	4	4	5	5	4	4	4	3	2	2
133	1	2	3	4	3	4	2	3	3	4	3	3	2	4	2	2	3	3
134	4	4	4	4	4	4	4	5	4	1	4	2	4	4	3	5	4	4
135	5	5	5	5	5	5	5	5	5	5	5	3	5	5	5	4	4	4
136	5	5	4	5	5	5	3	4	3	4	3	4	4	5	4	5	5	5
137	5	5	4	5	4	3	3	4	3	4	3	3	4	4	4	4	4	4
138	4	4	4	4	4	3	4	3	4	4	5	4	3	3	4	4	4	4
139	5	5	5	5	5	4	3	4	5	4	5	2	5	4	3	4	4	4
140	5	4	4	4	5	3	3	2	4	3	4	3	4	3	4	2	2	3
141	5	5	4	5	5	4	4	4	5	2	5	3	4	5	5	4	4	4
142	5	5	5	5	5	4	3	5	5	4	4	4	5	5	5	4	4	3
143	4	4	3	3	3	2	2	2	5	5	4	4	3	3	3	3	3	3
144	5	4	5	3	4	3	3	2	4	4	5	5	5	4	2	3	3	3
145	4	4	4	4	4	4	4	3	4	3	4	4	4	4	3	3	4	4
146	4	4	4	4	4	4	3	3	5	5	5	4	4	4	4	3	3	3
147	5	5	5	5	5	4	4	4	4	3	4	3	5	4	5	4	4	4
148	4	5	4	5	3	5	4	5	5	4	5	4	5	5	4	4	5	4
149	5	5	4	5	4	4	5	4	5	4	5	5	4	5	3	4	5	5
150	4	5	4	5	5	4	3	3	5	4	5	4	5	5	5	4	4	4
151	4	4	4	4	4	4	4	4	5	5	5	5	5	4	4	5	5	5
152	4	4	4	5	4	3	4	4	4	4	4	4	4	4	4	4	4	4
153	5	4	3	4	5	4	2	3	5	4	4	4	3	3	3	4	4	3
154	4	4	4	4	4	4	4	4	4	4	4	4	4	4	4	4	4	4
155	5	4	5	5	5	2	3	2	5	5	4	3	4	5	4	3	3	3
156	5	5	5	5	5	5	5	5	5	5	5	5	5	5	5	5	5	5
157	4	4	4	4	3	2	4	3	4	3	5	4	2	4	3	4	5	4
158	4	3	3	4	4	3	4	4	3	3	4	5	1	3	3	4	5	4
159	4	4	4	4	4	3	2	3	4	3	4	4	3	4	4	3	3	3
160	4	4	4	4	4	4	4	4	4	4	4	4	4	4	4	4	4	4
161	4	4	5	5	4	4	4	3	4	4	4	4	4	5	4	3	4	4
162	4	4	4	4	4	4	4	4	4	4	4	4	5	4	5	4	4	4
163	4	4	4	4	4	4	4	3	4	4	4	4	4	4	4	4	4	4
164	4	4	4	4	4	4	3	4	5	5	5	4	3	3	4	4	4	4
165	4	5	4	4	4	4	4	4	5	4	5	4	4	4	4	4	3	4
166	5	5	5	5	5	4	3	4	5	5	5	5	5	5	5	4	4	4
167	4	4	3	4	3	3	3	3	4	3	4	3	3	4	4	3	3	3
168	4	5	4	5	5	4	3	4	5	4	5	4	4	4	4	4	4	4
169	5	5	5	5	5	4	3	3	4	3	4	3	3	4	3	3	3	3
170	4	4	4	4	4	3	4	4	4	2	4	2	4	4	4	4	4	4
171	5	5	5	5	5	5	4	4	5	4	5	5	5	5	4	4	4	5
172	4	4	3	4	5	3	2	3	5	5	5	3	4	4	5	3	4	3
173	4	4	4	4	4	4	4	4	4	3	3	3	4	4	4	4	4	4
174	4	4	5	5	5	4	4	4	4	4	4	3	5	5	4	4	4	4
175	4	5	4	5	5	2	2	3	5	5	5	5	4	5	4	4	4	4
176	5	5	5	5	4	4	3	4	5	5	5	4	2	3	4	4	4	4
177	4	4	4	4	4	4	4	4	4	4	4	4	4	4	4	4	4	4
178	4	4	3	4	3	4	4	2	5	5	5	4	4	4	4	3	3	4
179	3	3	3	4	4	3	3	3	4	4	4	3	3	3	4	3	3	3
180	4	4	5	4	3	3	4	3	5	5	4	4	4	5	4	3	4	4
181	4	4	4	4	2	2	3	3	3	2	2	2	3	2	2	2	3	3
182	4	4	4	5	3	4	3	3	5	4	4	4	3	3	3	4	4	4
183	4	4	4	3	3	4	4	4	5	5	5	5	4	4	4	5	5	5
184	5	5	4	5	5	3	3	2	4	4	4	4	4	4	3	3	3	3
185	5	5	5	5	5	5	5	5	5	5	5	5	5	5	5	5	5	5
186	5	5	5	4	5	2	3	2	4	4	4	3	3	3	3	3	3	2
187	4	4	4	5	3	2	4	3	5	4	4	3	4	3	2	3	3	3
188	5	5	5	4	4	3	3	4	5	5	5	5	5	5	4	3	4	4
189	4	4	5	4	2	5	4	3	5	3	5	4	3	2	4	4	4	2
190	4	4	4	4	4	3	4	4	4	4	4	3	4	4	4	3	3	4
191	5	5	5	4	5	3	2	3	5	5	4	4	5	5	4	3	3	3
192	4	4	4	3	4	4	3	4	4	2	3	1	5	5	5	3	4	4
193	4	4	4	4	4	4	3	3	4	2	3	2	4	4	4	3	3	3
194	3	3	4	4	4	3	2	3	5	2	4	2	3	3	3	3	3	3
195	4	4	4	4	4	5	4	4	5	4	4	3	3	3	3	5	5	5
196	4	3	5	4	4	4	4	4	4	4	5	5	4	4	4	4	4	4
197	5	4	5	4	4	4	4	5	5	4	5	3	4	5	4	5	4	5
198	4	4	4	5	4	4	5	4	5	4	5	4	5	4	5	5	3	4
199	4	4	4	5	4	3	4	4	5	5	5	4	4	4	3	5	4	4
200	4	4	4	5	4	4	3	4	5	5	5	4	4	4	4	4	4	4
201	4	4	5	4	5	4	4	5	5	5	4	4	4	5	4	5	5	4
202	5	4	5	5	4	5	3	5	5	4	4	3	5	4	5	5	5	5
203	5	4	4	4	5	4	3	4	3	4	3	4	5	4	4	4	4	5
204	5	4	5	5	4	4	5	5	5	4	5	5	4	5	4	5	4	5
205	5	4	4	3	3	5	4	3	5	4	5	5	4	4	4	4	4	4
206	5	4	4	4	4	3	2	4	5	4	5	4	5	5	4	4	5	4
207	4	4	5	5	4	5	4	5	5	4	2	3	4	5	4	5	4	4
208	5	4	5	5	4	4	4	4	5	5	5	4	4	5	5	4	4	5
209	5	4	4	4	3	4	5	4	4	4	5	4	4	5	3	4	5	5
210	5	4	5	5	4	2	3	4	4	3	4	2	4	4	5	4	5	4
211	5	4	5	4	5	3	4	4	5	5	5	4	5	5	4	5	5	5
212	5	4	4	4	5	3	3	3	5	4	5	4	3	3	4	4	4	4
213	4	4	5	4	5	4	3	4	4	3	4	4	4	4	5	5	5	4
214	5	3	4	4	3	4	4	4	4	4	4	5	5	5	4	4	5	3
215	5	4	4	4	4	3	4	4	5	4	5	3	4	4	4	4	4	4
216	4	4	4	4	4	4	4	4	4	4	4	3	4	4	4	4	4	4
217	5	4	5	5	5	4	4	4	5	5	5	3	4	4	4	4	4	4
218	4	3	4	4	4	4	3	3	4	4	5	4	4	3	4	4	4	4
219	4	4	5	5	3	3	3	4	5	4	5	5	4	4	5	4	4	4
220	4	4	4	4	4	3	4	4	5	3	4	4	4	4	4	4	4	4
221	4	4	4	4	5	4	4	5	4	5	4	4	4	5	4	4	4	5
222	5	4	5	5	5	5	4	5	5	5	5	5	4	4	4	5	5	5
223	4	4	4	4	4	4	4	3	5	4	5	4	3	4	3	4	4	4
224	5	4	4	4	4	3	4	4	5	5	5	5	4	4	4	4	4	4
225	5	4	3	4	4	4	3	4	5	4	4	3	4	4	4	4	4	3
226	5	4	4	4	4	4	4	5	4	4	5	4	4	4	4	5	5	4
227	5	4	4	5	4	5	4	5	5	4	5	5	5	4	5	5	5	4
228	4	4	4	4	3	4	4	3	4	3	4	3	4	4	3	4	4	4
229	5	4	4	5	4	4	4	4	4	3	4	3	3	4	4	4	4	4
230	5	4	4	4	3	3	3	4	4	3	5	4	4	4	3	3	4	4
231	4	4	5	4	4	4	2	4	4	4	4	5	4	4	4	4	5	4
232	5	4	4	4	3	4	4	4	5	5	5	4	5	5	5	4	5	5
233	5	4	4	4	5	4	4	5	4	2	4	3	4	4	4	3	4	4
234	5	4	4	3	2	4	3	4	4	5	4	4	5	4	3	5	4	4
235	4	4	5	4	5	4	5	4	4	4	4	4	4	4	4	5	4	5
236	4	4	4	4	5	5	4	4	4	4	5	4	4	5	4	4	5	5
237	4	4	4	4	4	5	5	5	5	4	4	4	4	5	4	4	4	4
238	5	4	5	4	5	4	2	4	5	4	5	4	4	4	4	4	5	4
239	4	4	4	4	5	5	4	4	4	5	4	4	4	4	4	5	4	4
240	5	4	4	5	4	4	5	4	4	5	4	5	4	5	4	4	5	4
241	4	4	4	4	4	5	4	4	4	4	4	4	4	5	4	4	5	4
242	4	4	4	5	4	4	4	4	4	3	3	2	4	4	4	4	4	4
243	5	4	4	5	5	4	5	4	4	2	4	2	4	5	5	5	5	4
244	4	4	5	4	5	3	4	4	4	5	4	3	4	5	4	5	4	4
245	4	4	4	5	4	4	4	4	4	3	3	3	4	5	4	4	5	4
246	5	4	4	4	2	4	1	4	5	5	5	4	4	4	5	5	4	5

247	4	4	5	4	5	4	5	4	4	4	5	3	4	4	5	4	5	4
248	4	4	5	4	5	4	5	4	4	4	5	4	4	4	5	4	5	4
249	5	4	5	5	4	4	3	4	5	4	5	5	4	4	4	4	4	4
250	5	4	4	5	4	4	4	4	4	4	4	3	5	4	4	5	4	4
251	4	4	5	5	4	5	4	5	4	3	4	2	4	5	4	4	5	5
252	4	4	5	5	4	4	4	4	4	3	3	3	4	5	4	5	4	4
253	4	4	5	5	4	2	2	1	4	2	4	2	5	4	4	4	4	5
254	4	4	4	4	4	4	4	4	4	4	4	4	4	4	5	4	5	4
255	4	4	5	3	5	4	4	3	4	3	3	5	4	3	4	4	5	4
256	4	4	4	4	5	5	4	4	4	4	4	4	4	4	4	5	4	4
257	4	4	5	5	5	2	4	4	4	4	4	3	4	4	4	4	4	3
258	4	4	5	4	3	2	2	3	5	4	4	5	4	4	5	4	4	5
259	4	4	5	5	4	4	4	5	5	2	4	2	3	5	4	4	5	4
260	5	4	5	5	4	5	4	5	5	2	4	2	4	5	5	5	5	4
261	4	4	4	5	4	4	4	4	5	4	5	3	4	4	5	5	4	4
262	4	4	4	4	5	5	4	4	3	2	3	2	3	3	4	5	4	5
263	5	4	4	3	3	5	4	4	4	3	4	4	5	4	4	5	4	4
264	5	4	5	5	4	3	4	4	5	2	5	2	3	4	4	4	2	4
265	4	4	4	5	4	5	4	5	5	4	4	4	5	4	5	4	5	4
266	4	4	3	4	4	5	4	4	3	2	3	2	3	3	4	5	4	5
267	5	3	5	3	5	3	3	5	5	4	3	4	5	3	4	4	3	4
268	3	3	4	5	4	3	3	4	3	5	4	4	5	3	5	4	5	5
269	4	4	5	4	5	4	5	4	4	4	5	3	4	5	4	4	5	4
270	4	4	3	4	4	4	5	4	3	2	3	2	3	3	4	5	4	4
271	4	4	5	4	3	4	3	4	5	3	5	4	2	3	4	4	4	4
272	5	4	5	5	4	4	4	4	5	4	4	3	4	5	4	4	4	4
273	5	4	4	4	5	5	4	5	5	4	5	4	5	5	4	5	4	4
274	4	3	4	4	4	4	4	4	4	3	4	2	3	3	3	4	4	4
275	4	3	4	4	3	4	3	4	5	4	5	5	4	4	4	4	5	4
276	4	4	5	4	5	4	5	4	4	4	5	3	4	5	4	4	5	4
277	4	4	4	4	4	5	5	5	5	4	5	4	4	3	5	5	3	4
278	5	4	4	5	5	4	4	4	5	3	5	4	5	5	4	4	5	5
279	4	4	4	5	4	4	3	4	3	4	4	4	5	5	4	4	4	3
280	4	4	4	5	4	4	5	4	5	4	4	5	5	5	5	5	4	4
281	4	4	4	4	5	4	5	4	4	5	4	4	4	5	4	5	4	4
282	5	3	4	5	4	4	5	5	5	5	5	4	5	4	5	5	4	5
283	4	4	4	4	4	5	4	5	4	5	4	5	4	4	4	5	4	4
284	4	4	5	4	4	5	4	4	5	4	4	5	4	5	5	5	4	4
285	5	4	4	5	4	3	5	4	5	4	4	3	4	4	5	4	4	5
286	4	4	4	3	4	4	4	5	3	3	4	5	5	3	3	5	4	5
287	4	4	4	4	5	5	4	5	5	4	4	3	5	4	5	4	5	5
288	5	4	4	5	5	5	4	4	5	3	4	3	4	5	4	4	5	4
289	5	4	4	5	4	3	4	2	4	4	4	3	4	5	4	3	4	4
290	4	3	4	4	4	4	4	3	5	5	5	5	3	4	3	4	4	4
291	4	4	5	4	5	4	5	4	4	4	5	3	4	5	4	4	5	4
292	4	4	5	4	5	5	3	4	5	4	5	4	5	4	4	5	4	5
293	5	4	4	5	5	4	2	5	4	3	4	3	4	4	4	4	5	5
294	4	4	4	4	5	3	4	3	4	5	4	4	3	4	4	4	4	5
295	5	4	4	5	5	4	5	4	4	2	3	2	4	4	5	5	5	4
296	4	4	4	4	3	3	2	3	5	4	4	4	4	4	3	3	3	3
297	4	4	5	5	3	5	4	4	4	4	5	5	2	2	3	4	5	4
298	4	4	5	4	4	4	5	4	5	4	4	5	4	4	5	4	5	4
299	4	4	4	4	4	4	3	3	3	4	4	4	4	4	3	4	4	3
300	5	4	4	5	4	4	4	5	5	4	5	3	5	5	4	5	5	4
301	5	4	5	5	4	3	4	4	4	4	5	5	4	4	4	4	4	5
302	4	4	4	5	4	3	4	3	5	4	4	5	5	5	3	3	2	3
303	5	4	4	4	5	3	3	3	5	3	4	4	4	4	4	4	4	4
304	4	4	5	5	5	5	3	4	3	3	4	5	5	4	4	3	4	4
305	4	3	5	4	4	5	4	4	5	2	4	2	4	5	4	4	4	5
306	4	4	3	4	3	4	3	4	5	4	4	4	4	5	4	4	4	4
307	4	3	4	5	4	2	2	1	4	4	4	3	4	3	4	3	2	2
308	4	4	5	4	4	4	4	4	5	5	5	4	4	4	4	4	4	5
309	5	4	5	5	4	5	5	5	5	5	5	4	5	4	5	5	5	5
310	4	4	5	5	4	4	4	5	5	4	5	4	5	4	4	5	5	4
311	5	4	5	4	5	5	5	5	5	4	4	4	5	4	4	4	4	4
312	5	4	4	5	4	4	5	4	4	4	5	4	5	5	5	4	5	5
313	4	3	4	5	4	3	4	4	4	4	4	3	4	4	4	4	4	4
314	4	4	4	4	4	4	4	4	4	4	5	5	4	4	4	4	4	4
315	5	3	5	5	5	5	5	5	5	5	5	5	4	4	4	4	4	4
316	5	4	5	4	4	4	4	4	4	3	4	2	4	4	5	5	5	4
317	4	4	4	4	5	4	4	5	4	5	4	5	4	5	4	4	5	4
318	5	4	5	4	3	4	4	4	4	5	4	4	4	4	3	4	5	4
319	4	4	4	4	4	4	4	4	4	4	4	4	4	5	5	5	4	5
320	4	4	4	5	5	4	4	5	4	4	5	4	5	4	4	4	5	4
321	4	4	5	4	4	4	5	4	5	4	4	5	4	4	5	4	5	4
322	4	4	4	4	5	3	3	4	4	4	4	2	4	5	3	4	5	4
323	5	4	5	4	3	4	2	4	5	4	5	5	5	4	5	4	5	5
324	5	4	5	5	4	4	3	4	5	5	5	4	4	4	4	4	4	4
325	4	4	5	4	3	4	5	4	5	4	4	5	4	5	5	4	3	4
326	4	4	3	4	3	5	5	5	5	4	4	4	3	3	3	3	3	3
327	4	4	4	4	4	4	3	3	4	3	3	2	3	4	3	3	4	4
328	4	4	4	4	4	5	5	5	4	5	5	4	4	4	4	5	4	4
329	5	4	5	5	5	5	5	5	5	5	5	5	5	5	5	5	5	5
330	4	3	4	5	3	4	5	4	4	2	4	4	3	3	4	4	5	4
331	5	4	4	5	5	2	3	2	4	4	4	4	4	4	5	2	3	3
332	5	4	5	5	4	4	4	4	5	4	5	5	4	4	4	4	4	4
333	4	4	4	5	3	3	4	3	5	5	5	5	4	4	4	4	4	4
334	5	4	5	5	5	4	4	4	5	5	5	5	5	5	4	4	4	4
335	5	4	5	5	4	4	4	4	5	5	5	4	5	4	4	4	4	4
336	4	4	5	4	5	4	3	3	4	4	3	3	4	4	4	3	4	4
337	4	4	4	5	4	4	4	4	4	2	4	2	3	4	4	4	4	4
338	4	4	5	5	4	4	4	3	4	4	4	3	4	5	4	4	4	4
339	4	4	4	4	5	4	5	4	4	4	5	4	5	4	5	5	4	5
340	4	4	4	4	4	4	3	4	5	4	4	4	4	5	5	4	4	4
341	4	4	4	3	3	4	2	3	5	3	4	3	5	5	4	4	4	3
342	4	4	5	5	4	4	4	4	4	4	4	3	4	4	4	4	4	4
343	5	4	4	4	5	5	3	5	5	4	4	4	3	4	4	3	4	4
344	5	4	4	5	4	4	4	3	5	5	5	3	4	4	5	4	4	4
345	5	4	5	5	5	4	5	4	5	5	5	5	4	4	3	2	3	3
346	5	4	4	4	5	4	5	4	5	5	4	4	5	4	5	4	4	4
