# Supplementary material for: The media risk of infodemic in public health emergencies: Consequences and mitigation approaches
Source: PLoS One. 2024 Sep 12;19(9):e0308080. doi: 10.1371/journal.pone.0308080 (PMC11392340; doi:10.1371/journal.pone.0308080)
Supplement: S1 Appendix — (PDF) [file pone.0308080.s002.pdf]

Constructs and items

Dimension	Item	Resource
IA	(IA1) I will follow the development of the COVID-19 pandemic.<br>(IA2) When people around me discuss information about the COVID-19 pandemic , I try to learn about these issues from social media.<br>(IA3) I will follow the infection symptoms of the COVID-19 pandemic.<br>(IA4) I will be concerned about the precautionary measures taken by the government in response to the COVID-19 pandemic.<br>(IA5) I will pay attention to the supply of basic protective equipment (masks, protective clothing, disinfectants.	Xu (2020)<br>Powell D<br>（1996）
OL	(OL1) I think opinion leaders have relevant expertise.<br>(OL2) I think opinion leaders often respond to questions or topics from their fans.<br>(OL3) I think the information posted by the opinion leader is true.	Wang<br>（2018）
RP	(RP1) The COVID-19 pandemic has had a significant impact on the lives of residents.<br>(RP2) The COVID-19 pandemic has a high probability of widespread infection.<br>(RP3) The COVID-19 pandemic has serious impact on the economy and social order.<br>(RP4)The risk of the COVID-19 pandemic is difficult to control.	Slovic P<br>（1992）
ISW	(ISW1) After seeing information about the COVID-19 pandemic on social media, I feel obliged to tell as many people as possible.<br>(ISW2) I have a desire to retweet or tell others about the COVID-19 pandemic when it is updated.<br>(ISW3) I often discuss information about the COVID-19 pandemic with others.	Park J W,<br>Kwon O K,<br>Jang H Y et al<br>（2012）
PU	(PU1) I think the information on social media about the COVID-19 pandemic is useful.<br>(PU2) Information posted on social media helps me understand the COVID-19 pandemic more easily.<br>(PU3) Information posted on social media helps me better prepare for the COVID-19 pandemic .	Maltz<br>（2000）
