# Supplementary material for: The media risk of infodemic in public health emergencies: Consequences and mitigation approaches
Source: PLoS One. 2024 Sep 12;19(9):e0308080. doi: 10.1371/journal.pone.0308080 (PMC11392340; doi:10.1371/journal.pone.0308080)
Supplement: Attachment — Submitted filename: Reponse to Reviewers.docx [file pone.0308080.s003.docx]

Dear Editor and Reviewer,

Thank you for the opportunity to resubmit our manuscript “The media risk of infodemic in public health emergencies :Consequences and Mitigation Approaches” for consideration in PLOS ONE. We are grateful to you and the reviewer for the thoughtful comments and recommendations regarding the manuscript. In addressing them, we have made a large number of revisions based on your comments. With your help, the overall level of the paper has significantly improved compared to the initial manuscript. Thank you very much!

We have made significant revisions to key sections, which we have **highlighted in blue** in the attached document for your easy reference, focusing particularly on enhancing the clarity and precision of our arguments.

For ease of review, specific changes relevant to reviewer’ comments are highlighted in the manuscript.

Again, thank you for your consideration. We look forward to hearing your decision.

Sincerely,

Rui Shi, Xiaoran Jia, Yuhan Hu, and Hao Wang.

**Response to the Editor’s comments**

1. Please note that funding information should not appear in any section or other areas of your manuscript. We will only publish funding information present in the Funding Statement section of the online submission form. Please remove any funding-related text from the manuscript.

Author’s Response: Thank you so much!

We have already removed any text related to funding from the manuscript and input some of the information from the "Funding Statement" into the online form.

2. Please include your full ethics statement in the ‘Methods’ section of your manuscript file. In your statement, please include the full name of the IRB or ethics committee who approved or waived your study, as well as whether or not you obtained informed written or verbal consent. If consent was waived for your study, please include this information in your statement as well.

Author’s Response: Thank you so much!

We have incorporated an "Ethics Statement" in the Methods section of our paper, listing the approving institution: the Ethics Committee of Qinhuangdao First Hospital, Hebei Province, China. All participants signed informed consent forms prior to engaging in the study. (please see lines 207-210).

**Response to the Reviewer 1’s comments**

1. About the questionnaire application, how the sampling process was performed? How did the authors reach the number of 395 questionnaires to be sent? What was the population? I recommend the authors explain in more detail the data collection process, providing information related to these questions.
   Author’s Response: Thank you for your astute observation!

We have incorporated an exhaustive exposition on the sampling survey methodology within our paper. To guarantee representative sampling, this study utilized a hybrid snowball and random sampling strategy for questionnaire dissemination.The process is specifically divided into three steps. To guarantee representative sampling, this study utilized a hybrid snowball and random sampling strategy for questionnaire dissemination. The aim was to achieve demographic balance across gender, age, education, and internet usage.The detailed methodology is outlined as follows:surveys were distributed via stratified random sampling among various age and gender groups in the researchers' social networks, asking participants to haphazardly pass the questionnaire to their friends to fill out. Data collection from demographic categories that were properly represented was then stopped once a preset amount of responses were received. In order to reduce the possibility of sample biases, extra stratified random sampling was carefully carried out among underrepresented groups.Lastly, to counterbalance the limitations inherent in snowball sampling, supplementary random sampling was conducted in university libraries, enterprises, institutions, and outdoor parks. Respondents were selectively approached to enhance diversity, and questionnaires were thoroughly explained to ensure comprehension, thereby improving response quality and validity.This multi-faceted approach was instrumental in compiling a well-balanced and informative dataset for the study.

Furthermore, the questionnaire was released online and distributed through WeChat and Credamo sample pools. Credamo is an integrated smart research platform specializing in questionnaire design, sample provision, and statistical analysis. It streamlines data collection and analysis processes for academic and corporate users. Offering end-to-end services from questionnaire creation, online deployment, data gathering to statistical modeling, Credamo boasts a million-level online participant pool, ensuring data authenticity and diversity. It extensively supports universities, enterprises, and research institutions, facilitating efficient research project execution.(please see lines 236-249).

2. The authors stated that “The path analysis was carried out using AMOS 24.0 software …”. The software is an IBM product dedicated to Structural Equations modeling and analysis, so I recommend the authors provide in the Method section an introduction to IBM Amos 24.0 software, and the pipeline of the analytical process used. Note that the Data Analysis section describes this process in terms of results, so, the task here is to formulate, for instance, a flowchart of the whole analytical process, discriminating each step, in terms of methods used.
   Author’s Response: Thank you for your suggestions!

We have augmented the "Methods" section with a detailed explanation of the structural equation modeling approach and the specific process of model construction. (please see lines 192-199). In consideration of the logical structure of this paper, we have also included in the path analysis section a description of the AMOS 24.0 software usage, along with how this tool is employed to validate the path coefficients of the structural equation models,highlighting its capabilities in Structural Equation Modeling (SEM) and its relevance to our research.(please see lines 284-297). This paper endeavors to employ the AMOS 24.0 software, a prominent tool in SEM analysis, to calibrate and refine a theoretical model explicating the determinants influencing the information sharing willingness on public health emergencies.

3. Results and discussion are described in a “plain text” with no separation of subsections. I recommend the authors dedicate sections about theoretical and practical implications as well as the social consequences of their research. About the social consequences, I believe the paragraph started in line 402 of the manuscript (“Therefore, social media play an important role in the dissemination of information on public health emergencies. The adoption of suitable steps to avoid and contain the future deterioration …”) can be placed or be used as the base to create the new section.
   Author’s Response: Thank you for raising these important points!

We wholeheartedly agree with your suggestion to dedicate separate sections for discussing the theoretical and practical implications of our research, as well as its societal consequences. We have restructured the "Discussion" section, segregating it into distinct segments: one concentrating on the theoretical implications, another emphasizing practical applications, and a newly-added chapter devoted exclusively to examining the societal implications of our research findings.(please see lines 403-435).

Furthermore, we have relocated the paragraph started in line 402 of the original manuscript (“Therefore, social media play an important role in the dissemination of information on public health emergencies. The adoption of suitable steps to avoid and contain the future deterioration …”)to the Recommendations chapter, where we have built upon it to propose more targeted measures.(please see lines 437-464).

We believe this reorganization will significantly strengthen the narrative flow and emphasize the comprehensive significance of our work. We appreciate your guidance in this enhancement process and look forward to implementing these changes to elevate our manuscript's overall quality.

4. In Conclusion, it is important to clarify what were the limitations, difficulties, and challenges faced by the authors during the development of their study. These elements are relevant to ensure replicability. It is also important to point out some directions for new studies continuing or based on the study the authors developed.

Author’s Response: Thank you for your constructive feedback.

Thank you for your thoughtful remarks which have greatly contributed to enhancing the rigor and utility of our study. We fully concur with your perspective on the importance of acknowledging limitations, difficulties, and challenges encountered during our research, as these aspects are crucial for promoting methodological transparency and facilitating future replication attempts.

In light of your feedback, we intend to enrich our conclusion section by explicitly addressing the following points:First, the challenge lies in capturing data amidst the dynamism of social media, where the rapidly evolving landscape of information epidemics and the sheer volume of data hinder precise assessments of long-term impacts. Second, the complexity in measuring risk perception and information-sharing behaviors arises due to the diversity in individual psychology, subjective interpretations of risk, and inconsistencies stemming from varied cultural backgrounds and levels of media literacy, all of which limit the generalizability of the study findings.(please see lines 466-486).

**Response to the Reviewer 2’s comments**

1. Writing style and structure needs to be greatly improved for conveying the findings and takeaways of the work and before this work can be reviewed properly for its contributions.

Author’s Response: Thank you for your suggestions!

Regarding your suggestions on the style and structure of my paper, we have given them thoughtful consideration and carried out the necessary amendments. Throughout the revision process, we meticulously examined your review comments and also drew insights from dozens of articles published in the journal, striving to adjust the style and structure while preserving the integrity and accuracy of the content.

**In terms of style,** we have placed greater emphasis on clarity and logical coherence, striving to make the narrative of the paper more fluid and natural, thereby enhancing its comprehensibility. Additionally, we have reviewed and revised the technical terminology and expressions used in the paper to ensure their precision and consistency. For example, in the **Introduction** section, we have built upon the integration of the research background and recent studies to explicitly state the objectives and anticipated outcomes of our research.(please see lines 12-80). Within the section on **Hypotheses develpoment**, we have consciously refrained from delving too deeply into the theory of risk perception, instead redirecting the focus towards a meticulous presentation of each hypothesis. This shift ensures that the prime objectives and forecasted results of the study are conveyed with clarity, thereby enhancing readers' grasp of the central questions and the crux of our investigation. By doing so, a solid foundation is established for the following analytical examinations and discussions.(please see lines 81-189).

**As for the structure,** we realigned the sections and rearranged paragraphs based on your guidance, with the aim of better illustrating the logical relationships and argumentative progression within the paper. Efforts were also made to eliminate redundancy and repetitive content, rendering the paper more concise and compact. In the **Discussion** section, we uncovered a significant positive correlation between opinion leaders, information attention, and the willingness to share information, endorsing our hypotheses and highlighting the substantial influence of social media on information sharing. Furthermore, the incorporation of risk perception illuminated the intricacies of these relationships, demonstrating its mediating role in the link between opinion leaders, information attention, and the willingness to share. Lastly, the moderating effect of perceived usefulness on the relationship between information attention and sharing willingness was validated, further enriching our findings.(please see lines 327-401). In the **Conclusions**, this study theoretically enriches the understanding of the relationship between social media, risk perception, and the willingness to share information, offering a fresh perspective on their underlying mechanisms. Practically, our findings can serve as a basis for policymakers, enabling them to better comprehend and address pertinent issues.(please see lines 402-435). In the **Recommendations** section, we have put forth more specific measures, such as: enhance transparency and credibility, risk communication strategies, empower opinion leaders and influencers and research and monitoring.(please see lines 436-464).

2. Paper suffers from redundancy, with information repeated across different sections, making the paper much lengthier than it should be. Repetition also causes to dilute the key points of the work.
   Author’s Response: Thank you for your meticulous review and for pointing out the issue of redundancy in our paper.

We fully understand that unnecessary repetition can undermine the clarity and impact of our key findings. The original manuscript's literature review excessively emphasized risk perception, resulting in repetitive content. Additionally, the section overlappingly discussed the research hypotheses both in the literature review and conclusion, unduly extending the paper's length and clouding its primary focus. To tackle these issues, comprehensive revisions were executed as follows:

Firstly, we integrated repeated information originally included for reinforcement, eliminating superfluous explanations of concepts and methodologies to promote clarity and brevity.

Secondly, the introduction was enriched to encompass not only the research backdrop but also a synopsis of the latest developments within the discipline, facilitating readers' comprehension of the field's ongoing progress and existing gaps. This sets a pertinent context for the study.(please see lines 12-80).

Next, the conclusion was reoriented to concentrate specifically on how social media influences users' risk perception and, subsequently, their inclination to share information, staying aligned with the article's core subject matter. By pruning content that did not advance the central thesis directly, we intensified the focus on our pivotal arguments and discoveries.(please see lines 403-435).

Lastly, to uphold coherence and prevent redundancy when similar points arose in different contexts, these sections were either restated in fresh terms or cross-referenced to prior explanations, maintaining continuity without duplicating text verbatim.

3. The literature review should be streamlined by focusing on the most relevant studies and avoiding excessive detail and definitions of the underlying concepts.

Author’s Response: Thank you for your suggestions!

After meticulously reviewing your reviewer comments, we recognized the need to enhance the logical structure of the article. Consequently, we have incorporated discussions on the most recent research in the introduction, with the aim of enhancing the narrative's coherence and readability. Simultaneously, we have summarized and synthesized relevant literature, steering clear of unnecessary elaboration on fundamental concepts. Furthermore, in integrating these latest findings, we have delved into authoritative publications from recent times, meticulously analyzing the principal arguments put forth in these works.(please see Introduction 2-80). My objective was to integrate these latest findings closely with my research topic, thereby presenting a more comprehensive and precise depiction of the current dynamics and cutting-edge trends in the field. We are confident that the inclusion of these recent studies will facilitate readers' understanding of the latest developments in this domain and augment the academic worth and impact of the paper.

4. The flow of the paper needs major improvements. Sections and text feel disjointed, and the transitions are not clear. I struggled with making a connection between risk perception and information sharing behavior which are the key ideas of the paper.

Author’s Response: Thank you for your insightful feedback.

Having carefully read through your review report, we gained a profound understanding of the areas in which my paper required improvement regarding structure and argumentation.In response to your comments on the structure and organization of chapters, we have conducted thorough revisions. This round of modifications particularly focused on adjusting the chapter layout to better adhere to logic and academic standards.

Since structural equation modeling (SEM) typically relies on a theoretical framework or assumptions, in the section **Hypotheses Development**, we meticulously analyzed each hypothesis's independent and dependent variables, along with their direct or indirect effects, thereby enhancing the interpretability of our findings.(please see lines 81-189).

Within the **Research Method and Design** section, we first introduced the concept of SEM and presented the theoretical model guiding our study.(please see lines 191-205). To measure latent variables that cannot be directly observed, we employed a questionnaire survey method, transforming these latent variables into quantifiable observed data, and meticulously detailed the questionnaire design process.(please see lines 211-234). We then outlined the questionnaire distribution procedure, including pilot testing, the method of distribution, and the sampling process for the survey.(please see lines 235-262).

In the **Data Analysis section**, we initiated with reporting the results of reliability and validity tests.(please see lines 264-282). Following this, path analysis and hypothesis testing were conducted based on the established foundation.(please see lines 283-297). Lastly, we examined the mediating effect of risk perception and the moderating role of perceived usefulness.(please see lines 299-325).

The **Discussion** section thoroughly dissects the outcomes of each path analysis, transparently demonstrating whether the data supports these relationships, thereby validating our hypotheses. This approach contributes to rendering the paper more comprehensive and rigorous.(please see lines 327-401).

In the **Conclusion**, we underscored the unique contributions of our study and elucidated its theoretical and practical implications.(please see lines 402-435).

Pertinent **recommendations and strategies**, tailored to our research theme, were proposed.(please see lines 436-464).

Finally, we highlighted unresolved **limitations and issues** not considered in our current study, offering new avenues of research and methodologies for fellow researchers to explore.(please see lines 465-484).

Moreover, we dedicated additional effort to expand and delve deeper into the pivotal aspect – **the relationship between risk perception and information sharing.** During the revision process, we meticulously examined the intrinsic link between risk perception and information sharing, and, by incorporating relevant theories and the latest research findings, provided a thorough explanation and substantiation of this relationship.

For instance, in the **Introduction** section, risk perception, as a psychological factor, emerges as a pivotal determinant influencing users' information filtering, evaluation, and dissemination behaviors. Users actively search and filter information from a variety of sources in their capacity as information seekers, and they also serve as disseminators by forwarding content they find important to other members of their social networks[1]. Information screening, appraisal, and sharing behaviors are significantly influenced by psychological elements (e.g., fear, anxiety, and trust), the degree of attention paid to emergency information, and the subjective evaluation of information[2].

The **Hypothesis Development** section particularly delves into how opinion leaders and information attentionality leverage risk perception as a mediating variable to affect users' willingness to share information.

Meanwhile, in the **Discussion** section, empirical evidence confirms the positive impact of risk perception on the intention to share information, thereby reinforcing its pivotal role in this behavioral process. In times of crisis, there is a sharp increase in the public's demand for risk-related information due to their pressing need to remain up to date on the most recent advancements, preventive measures, treatment options, and other relevant information in order to make decisions that will protect their own and their families' well-being[3].

We aimed to uphold my arguments with clear logic and robust evidence, thereby enhancing the academic merit of the paper.

**References:**

[1] Shao, W., Hui, P. M., Chen, X., & Pan, Y. (2020). The role of social media in the transmission of COVID-19 information: Information cascade and misinformation. International Journal of Environmental Research and Public Health, 17(14), 5149.
[2] Douglas K M, Uscinski J E, Sutton R M, et al. Understanding conspiracy theories[J]. Political psychology, 2019, 40: 3-35.
[3] Li R. Analysis of the victims information behavior characteristics in sudden disaster context: A case study on the victims of 7.20 Zhengzhou metro flooding incident[J]. Information Studies:Theory & Application,2023,46(03):127-133.
5. The language seems to be convoluted and unduly complicated. It feels like I am reading translated text, as sentences seem to break midway followed by another idea or sentence. Additionally at some places, the sentences are too long don't seem to be conveying anything important.

Author’s Response:Thank you for raising these important points!

We have meticulously reflected upon the shortcomings in our paper and resolved to undertake a thorough re-polishing of the language, alongside adjusting the logical sequence of sentences. Fully aware of the crucial role that linguistic fluency plays in determining the quality of a paper, we have endeavored to refine sentence structures for enhanced clarity and precision. Throughout the revision process, each sentence was meticulously scrutinized to guarantee logical coherence and accurate expression. Additionally, we have paid close attention to fostering linguistic fluidity and coherence, thereby rendering the overall reading experience of the paper more natural and effortless.

6. The conclusion should be strengthened by emphasizing the unique contributions of the study, highlighting how the findings extend or augment the previous research and offer new perspectives on managing infodemics. The recommendations seem textbook version of existing suggestions for mitigating misinformation. The paper should include succinct but in-depth discussion of how these recommendations can be implemented effectively.

Author’s Response: Thank you for your suggestions!

We fully acknowledge the importance of emphasizing the unique contributions of our study in the conclusion. To address this, we revised the conclusion section to underscore precisely how our findings build upon and differentiate themselves from prior research, shedding fresh light on the management of “infodemics”.(please see Conclusions).

**Theoretically**, this research enriches our understanding of the complex dynamics between technology use, particularly social media platforms, and human behavior in times of crisis. By delving into the cognitive and psychological processes that underpin risk perception and information dissemination, it contributes to the advancement of communication theories, including infodemiology, risk communication, and social amplification of risk frameworks. It also elucidates the role of opinion leaders in shaping public discourse and highlights the significance of individual differences in information processing as critical moderators in risk communication, thereby refining our knowledge of information behavior in the digital age.(please see lines 406-414).

**Practically**, the study holds significant value for policymakers, public health officials, and crisis management teams. Insights gained can inform the development of more targeted and effective communication strategies aimed at mitigating the negative effects of infodemics. Understanding how social media shapes risk perceptions can guide the creation of tailored messaging that enhances public trust, combats misinformation, and promotes responsible information sharing. Moreover, by identifying the unique contributions of opinion leaders and the impact of user information processing styles, the research offers actionable guidance for leveraging influential voices in social networks to disseminate accurate and timely information, fostering a more resilient and informed society.(please see lines 415-424).

This research makes unique contributions by integrating multi-disciplinary perspectives to provide a comprehensive analysis of the infodemic landscape. It moves beyond descriptive analyses to offer a nuanced examination of the causal pathways linking social media exposure, individual cognition, and collective behavior.By highlighting the intricate interplay among social media environments, risk perception, and information sharing, it underscores the necessity for a comprehensive, bidirectional approach to risk communication that addresses not only the spread of misinformation but also fosters an ecosystem conducive to the accurate and responsible exchange of information.Ultimately, this work paves the way for evidence-based interventions that can effectively navigate the challenges posed by infodemics in future public health emergencies.(please see lines 425-435).

Regarding the recommendations, we understand your concern that they may appear overly generic at present. To address this issue, we have incorporated specific and actionable strategies, delving deeper into four key aspects, with a focus on elucidating their context-specific nuances and potential challenges. This approach ensures that our recommendations not only hold theoretical validity but are also practicably feasible within real-world scenarios.(please see lines 436-464).

7. The paper's central theme seems to drift towards related but tangential topics, such as the general impact of social media on information dissemination, please try to maintain a sharper focus on the core theme.

Author’s Response: Thank you for your astute observation!

We appreciate your feedback highlighting the need for a sharper focus on the paper's central theme. The original manuscript suffered from an unclear logical structure and imprecise expression in its narration, which obscured the central theme of the article.

In light of your comment, we have committed to revising the manuscript to ensure that it stays meticulously aligned with the core topic, which is the mechanism by which social media shapes users' risk perception and information sharing willingness amidst public health emergencies.For example, in the **Discussion** section, a comprehensive examination of the impact of social media on the willingness to share information was conducted.(please see lines 328-363).

Within the social media landscape, the actions of opinion leaders intertwine with users' levels of information attention, collaboratively shaping patterns and intensities of information dissemination. Leveraging their authority, emotional resonance, and agenda-setting capabilities, opinion leaders steer user focus towards specific information, while elevated attention, in turn, amplifies the desire to share, fostering a feedback loop from attention to dissemination. The dual challenges of information overload and misinformation underscore the growing reliance on opinion leaders for content curation and validation, as well as the need for individual discernment in processing information prior to sharing decisions.In conclusion, the dynamics of information sharing within social media ecosystems emerge as a product of the interplay between the sway of opinion leaders and the level of attention accorded to information. This intricate relationship exerts profound implications for the efficiency, reach, and societal impact of information diffusion, offering a pivotal lens through which to comprehend the intricacies of the information ecology.

Moreover, in alignment with the theme of the article: Social media play an important role in the dissemination of information on public health emergencies, we have removed or condensed discussions that might divert attention away from the central argument. Particularly, in the Recommendations section, in light of the current information epidemic, it's critical to comprehend the media's fundamental role in public health catastrophes as well as the intricate ecology of information epidemics. The governance of the information epidemic, being a multi-risk phenomenon shaped by the mediated influence of emergencies, must take into consideration the interaction between the media and emergencies, build a human-centered discourse system and its guiding mode of emergency management, and then pertinent countermeasures have been put forward.(please see lines 436-464).

8. For data collection, how were the target poulation selected. Moreover, most of the survey takers are highly educated which obviously creates a bias in the analysis.

Author’s Response: Thank you for raising these important points!

Regarding the selection of the target population for data collection, we have revised the methodology section to provide a clearer explanation of the participant selection process, including the criteria and any limitations. Participants were identified using a hybrid snowball and random sampling strategyto ensure representation across different demographic segments. The method involved distributing surveys via stratified random sampling across age and gender groups in social networks.(please see lines 236-249).

Regarding the issue of the majority of survey participants having higher levels of education, due to an inaccurate previous portrayal, we have revised the educational background of our survey participants, clarifying that the majority of the surveyed individuals are undergraduates, constituting 42.45%, followed by those with vocational college education, representing 38.79%.(please see lines 256-262).

Considering the imprecision in previous descriptions, we have revised the educational background details of our surveyed cohort, explicitly stating that the majority of respondents hold educational qualifications at the vocational or undergraduate level. Of the previously mentioned 90.17% of surveyed individuals possessing tertiary or higher education, 38.79% were from vocational colleges, 42.45% were bachelor's degree holders, and 8.93% had postgraduate or higher qualifications. Amidst China's continually advancing education system, proliferating educational resources, and society's broad emphasis on higher education, this composition contributes to a general elevation in national educational attainment. In contemporary China, where vocational and undergraduate education are extensively disseminated, postgraduate and higher qualifications distinguish an elite educational stratum.

Furthermore, aligning with our sample's educational profile, the 53rd Statistical Report on China's Internet Development issued by CNNIC (China Internet Network Information Center) reveals an ascending trend in the internet literacy levels among Chinese netizens, reaching a fundamental proficiency threshold. This trend resonates with the educational composition observed in our study.

Our stratified sampling method, it should be noted, was not explicitly designed around educational attainment but instead focused on the more overt and readily distinguishable demographic dimensions: "gender" and "age." By stratifying according to these two criteria, we aimed to facilitate comprehensive coverage across diverse age groups and social segments, which inherently led to a concentration of participants with elevated educational backgrounds. This approach underscores our dedication to obtaining a representative sample of the population within the practical constraints of sampling logistics.

Lastly, individuals with higher levels of education tend to exhibit enhanced analytical and judgment capabilities. In the context of public health emergencies, they are potentially more adept at filtering, evaluating, and disseminating valuable information. Conversely, we posit that the high-education demographic is better equipped to articulate their thoughts clearly, providing accurate and comprehensive information, thereby facilitating the acquisition of data that is both high-quality and reliable.

9. Please try including informative figures to present key findings and helpwith understanding and engagement of the reader. The included figure on moderated mediation was confusing as well.

Author’s Response: Thank you for your constructive feedback.

We completely agree that incorporating informative figures can greatly enhance the presentation of key findings and facilitate reader comprehension and engagement. In response to your suggestion, we have added several illustrative figures, including graphs, charts, and diagrams, to visually represent the core data and trends uncovered in our research. For instance, to more clearly illustrate the model fit, **Table 3** has been added. To explicitly depict the relationships among various paths and validate hypotheses, **Figure 2** has been replaced and an additional table, **Table 4**, presenting the results of the study hypotheses, has been incorporated. To facilitate understanding of the mediation effect, an explanation highlighting the role of risk perception as a mediator in both pathways has been included.(please see lines 299-300). Furthermore, to more clearly demonstrate the moderating impact of perceived usefulness, **Figure 3** has been introduced.(please see lines 315-326). These enhancements aim to provide readers with a more coherent and logical presentation of our findings.

Each figure will be accompanied by a clear caption explaining its content and relevance to the study, ensuring that they augment rather than replace the written content.Specifically, the figures illustrating the moderating and mediating effects have been revised and improved.(please see Meditating effect and Moderating effect lines 299-326).
