# Supplementary material for: The media risk of infodemic in public health emergencies: Consequences and mitigation approaches
Source: PLoS One. 2024 Sep 12;19(9):e0308080. doi: 10.1371/journal.pone.0308080 (PMC11392340; doi:10.1371/journal.pone.0308080)
Supplement: Attachment — Submitted filename: Reponse to reviewers.docx [file pone.0308080.s004.docx]

Dear Editor and Reviewer,

Thank you for the opportunity to resubmit our manuscript “The media risk of infodemic in public health emergencies : Consequences and Mitigation Approaches” for consideration in PLOS ONE. We are grateful to you and the reviewer for the thoughtful comments and recommendations regarding the manuscript. In addressing them, we have made a large number of revisions based on your comments. With your help, the overall level of the paper has significantly improved compared to the initial manuscript. Thank you very much!

We have made significant revisions to key sections, which we have **highlighted in blue** in the attached document for your easy reference, focusing particularly on enhancing the clarity and precision of our arguments.

For ease of review, specific changes relevant to reviewer’ comments are highlighted in the manuscript.

Again, thank you for your consideration. We look forward to hearing your decision.

Sincerely,

Rui Shi, Xiaoran Jia, Yuhan Hu, and Hao Wang.

**Response to the Reviewer 1’s comments**

Dear Reviewer,

We sincerely express our gratitude for the reviewer's demonstration of professional rigor and a high sense of responsibility towards academic pursuits. Your endorsement of our preliminary revision efforts is greatly honored. In response to your subsequent insightful comments and observations, we have meticulously revised and fine-tuned the manuscript, ensuring that all concerns have been satisfactorily addressed.

Please review the attached detailed responses to your concerns, along with the revisions we've carefully made to address each point you've raised.

1. How does the analysis of the infodemic scenario contribute to assisting public health authorities in developing strategies capable of mitigating the stress effects of these situations? What technological tools can be used?For example: how can psychometrics be used in these situations?

Author’s Response: Thank you for your astute observation!

In response to the suggestion to incorporate a discussion on the utilization of psychometrics to assist public health authorities in devising strategies to mitigate the stress effects of misinformation, we have revised the manuscript accordingly: Social media play an important role in the dissemination of information on public health emergencies. The implementation of appropriate measures to prevent and mitigate the escalation of public health crises is significantly enhanced by access to accurate and factual information. This enables individuals to develop a realistic understanding of the risks involved and gauge the seriousness of such emergencies accurately. However, the proliferation of false information can lead to heightened fear and panic, causing individuals to misinterpret the severity of the situation and adopt harmful behaviors based on misinformation. This not only undermines public health efforts but also takes a toll on mental health. By understanding the dynamics of information spread, public health authorities can develop targeted interventions. (please see lines 448-457).

Psychometric analysis has been incorporated into the specific measures. Utilizing psychometrics enables the identification of vulnerable populations who may be more susceptible to misinformation and its associated stress effects. By understanding the psychological profiles of individuals prone to anxiety and misinformation acceptance, targeted interventions can be designed. These might include mental health support services, fact-checking resources, and community-led initiatives that foster critical thinking and resilience. (please see lines 471-476). The revised section highlights the importance of psychometric analysis in understanding the cognitive and emotional responses of individuals to misinformation.

2. Finally, I recommend that the authors conduct a general review of the use of the English language. I found, for example, the use of the compact form “it’s” when it is formally recommended to use “it is” (see line 442). There is a single occurrence of this use, from what I found through a search throughout the text, however, I strongly recommend a general revision to leave the text in a "standard" format for English usage.

Author’s Response: We would like to express our sincere gratitude for the reviewer's meticulous attention to detail and constructive feedback regarding the usage of the English language in our manuscript.

In accordance with your insightful recommendation, we have embarked on a comprehensive examination of the entire manuscript to ensure conformity with standard language usage. Particularly, concerning the issue you pinpointed at line 442 involving the casual form "it's", we have taken corrective action.

Moreover, taking the reviewer's advice to heart, we have decided to conduct a thorough review of the entire text to ensure that all instances conform to standard English usage. For instance, in the Introduction section, the original expression was "Gunther Eysenbach first proposed the idea of infodemiology in 2002 with the goal of examining the distribution and determinants of correct and incorrect information related to public health, directing patients and experts in the area toward reliable, accurate information", which has been revised to "Gunther Eysenbach initally proposed the term of infodemiology in 2002, aiming to investigate the distribution and determinants of health-related information, both accurate and erroneous, guiding patients and professionals in the field toward trustworthy and precise information". (please see lines15-19). We have also revised the expressions in the Introduction and Hypotheses development. This will include a careful examination of grammar, syntax, and style to eliminate any casual language or colloquialisms that may inadvertently deviate from formal academic writing conventions.

We appreciate the reviewer's guidance in helping us improve the quality of our submission. We look forward to incorporating these changes and resubmitting a polished and refined version of our paper for your consideration.

**Response to the Reviewer 3’s comments**

Dear Reviewer,

We are immensely grateful for the time and expertise you are dedicating to reviewing our manuscript. We are aware that you might be evaluating the original version of our manuscript. It's important to note that a revised version has already been developed, thoroughly addressing the concerns raised by previous reviewers. Building upon this improved draft, we have now incorporated further amendments in direct response to your suggestions, ensuring that our manuscript is finely tuned to meet the high standards of scholarly discourse.

Enclosed below are our detailed responses to your concerns, alongside the modifications we've made in response to your valuable feedback.

1. The English of this manuscript definitely needs to be polished or rechecked at least. For example, in lines 111 and 307, "Crown pneumonia epidemic" and "new crown outbreak" seem to refer to COVID-19, but these expressions are completely incorrect in English.

Author’s Response: Thank you for your careful reading of our manuscript and for pointing out areas where the English language usage can be improved. We appreciate your attention to detail and acknowledge that the expressions "Crown pneumonia epidemic" and "new crown outbreak," as noted in lines 111 and 307, were indeed imprecise and non-standard in English.

In response to your feedback, we have taken the following actions:

1. We have corrected the inaccurate phrases to "COVID-19" respectively, ensuring that our terminology aligns with the accepted English nomenclature.
2. Beyond addressing the specific issues you highlighted, we have also engaged a native English-speaking professional editor to review and polish the entire manuscript. This thorough revision aims to enhance the clarity, coherence, and overall standard of English throughout the text.
2. Please describe the process of distributing the questionnaires in more detail, including the characteristic of the respondents. For example, were the questionnaires distributed online or offline? Was the distribution process random? Did the respondents belong to a specific group or organization? Explain any potential biases in the data collection process and how these biases were controlled.

Author’s Response: Thank you for your insightful feedback.

Between March 1 and March 7, 2024, the questionnaires were released online through WeChat and the Credamo([https://www.credamo.com/home.html)](https://www.credamo.com/home.html) platform, aiming to maximize accessibility and inclusivity by reaching out to a diverse participant base digitally. (please see lines 241-243).

To guarantee the representativeness of the research sample, this study adopted an integrated sampling strategy that artfully combined snowball sampling with random sampling methods. This approach aimed to construct a sample framework that accurately reflects the structure of internet users, particularly in terms of gender, age, and educational background. The detailed methodology is outlined as follows: surveys were distributed via stratified random sampling among various age and gender groups in the researchers' social networks, asking participants to haphazardly pass the questionnaire to their friends to fill out. Data collection from demographic categories that were properly represented was then stopped once a preset amount of responses were received. Lastly, to counteract the bias of snowball sampling, targeted random distribution of questionnaires was conducted on the Credamo platform for underrepresented groups. Respondent selection enhanced sample diversity, while accompanying detailed questionnaire explanations secured participant understanding, boosting response quality and data validity, and solidifying research reliability. This multi-faceted approach was instrumental in compiling a well-balanced and informative dataset for the study.(please see lines 244-259).

The respondents were not confined to any specific group, the age range and educational backgrounds of the participants were notably wide, encompassing individuals from various life stages and academic achievements, which showcase the breadth and diversity of the survey population. (please see lines 264-273).

Potential biases in the data collection process were carefully considered and addressed. For instance, to mitigate response bias, we ensured the anonymity and confidentiality of the participants, encouraging honest and unbiased responses. Additionally, we took measures to reduce sampling bias by reaching out to underrepresented groups and employing snowball sampling techniques when necessary. (please see lines 253-268).

3. Provide more detail on the demographic characteristics of the sample and justify the sample size.

Author’s Response: We appreciate your suggestion to provide more detail on the demographic characteristics of our sample and to further justify the sample size.

In response to your comment, we have revised the "Sample and data collection" section of the manuscript to include a more comprehensive breakdown of the demographic variables of our sample. Specifically, we have added detailed information about the gender, age distribution, and educational background of the participants. This additional information will help readers better understand the composition of our sample and its relevance to the research questions. (please see lines 264-273 and Table 1.).

Regarding the sample size justification, for exploring variable relationships via structural equation modeling, a minimum sample size of 200 is advisable, ideally exceeding ten times the number of observed variables for robust analyses [1]. In this study, 395 questionnaires in total were sent; 346 of those were deemed legitimate, and the effective recovery rate was 87.59%. (please see lines 260-263).

**References:**

[1] Bentler P M, Chou C P. Practical issues in structural modeling[J]. Sociological methods & research, 1987, 16(1): 78-117.

4. The methodology section lacks details on the questionnaire development process. How were the items selected or adapted from existing scales? Were any pilot studies conducted to assess the validity and reliability of the questionnaire?

Author’s Response: We sincerely appreciate the reviewer's thorough evaluation and insightful comments regarding the methodology section of our manuscript.

To address the reviewer's concerns, we have revised the manuscript to include a detailed description of the questionnaire development process. (please see lines 206-233).

We drew upon established scales (please see lines 206-212) and adapted certain items to align with the specific objectives of our study, ensuring that the questionnaire captured the multifaceted nature of information dissemination in the context of health crises. In concordance with the study hypotheses and mindful of the exigencies dictated by the COVID-19, a meticulous revision and contextualization of survey items sourced from antecedent investigations was undertaken. Precisely, within the rubric measuring information attention, pandemic-specific nuances were integrated, exemplified by the item reflecting vigilance over the evolving dynamics pertinent to the COVID-19. Concerning risk perception, the awareness of potential perils intrinsic to the COVID-19 was emphasized through the calibration of relevant questions. Furthermore, in the evaluation of proclivity towards information sharing willingness, the pertinence of shared information to the COVID-19 milieu was explicitly delineated, as embodied in the item highlighting the propensity to disseminate or alert others upon receipt of updated the COVID-19 information. Through these targeted refinements, the questionnaire's contemporaneity and situational specificity were assured, thereby facilitating a more accurate delineation of participant behavioral paradigms and psychological orientations situated within the COVID-19 framework. (please see lines 212-225).

To assess the validity and reliability of the questionnaire, we conducted a pilot study involving a sample of 104 participants who were demographically similar to our main study population. The pilot study enabled us to evaluate the clarity, comprehensibility, and relevance of the items, as well as to test the internal consistency and construct validity of the questionnaire. Based on the feedback received and statistical analyses, minor adjustments were made to refine the wording of some items and to ensure that the questionnaire was both valid and reliable for measuring the constructs of interest. Furthermore, we utilized Cronbach's alpha to measure the internal consistency of the scale, which yielded satisfactory results above the conventional threshold of 0.7, indicating that the items within each subscale were sufficiently correlated to measure the intended constructs reliably. (please see lines 234-239).

5. Using the words “Cronbach's alpha coefficient” or “Cronbach's α” instead of “Cronbach's”.

Author’s Response: We are grateful for the reviewer's meticulous attention to detail in our manuscript.

We have since reviewed the entire manuscript and have rectified this inconsistency. Now, wherever applicable, we have used the "Cronbach's α" to refer to the measure of internal consistency. (please see lines 281).This change ensures that our terminology is scientifically accurate and consistent with the conventions of psychological and statistical research.

6. The authors briefly introduce the aim of the study in the abstract. However, I suggest to provide a more detailed study aim in the introduction section.

Author’s Response: We appreciate the reviewer's thoughtful feedback regarding the clarity and depth of our study's aims as presented in the abstract and introduction sections.We recognize the importance of clearly articulating the research objectives to provide a solid foundation for the study and guide readers' expectations.

Following the reviewer's suggestion, we have revised the abstract to offer a more detailed and explicit statement of the study's aims. The revised abstract now highlights the specific gaps in knowledge that our research seeks to address and outlines the primary research questions and hypotheses we intend to explore. This enhanced clarity will help readers immediately grasp the purpose and significance of our study. (please see lines 66-78).

Additionally, we have expanded the introduction section to provide a comprehensive rationale for our research. We have included a detailed discussion of the background, including a review of the existing literature, which underscores the need for our investigation. We have also delineated the specific objectives of our study, explaining how they contribute to the broader field and what new insights we anticipate our research will provide. (please see lines 2-65).

7. Discuss potential limitations of the study in more detail, including any methodological constraints and generalizability issues.

Author’s Response: We are grateful for the reviewer's constructive suggestions regarding the discussion of our study's potential limitations.

In response to the reviewer's suggestion, we have expanded the "Research limitations" section of our manuscript. We have now provided a more detailed and nuanced discussion of the potential limitations of our study, and suggested avenues for future research that could address these constraints.

Specifically, we have highlighted the following points:

Primarily, the intrinsic dynamism and perpetual evolution of social media platforms, alongside the resultant information ecosystems, present a formidable challenge to encapsulating a stable snapshot of the infodemic landscape. The empirical research methodology deployed herein, while advantageous in illuminating phenomena and relationships at specific temporal junctures, falls short in tracing and comprehending the longitudinal trends and underlying mechanisms of information sharing intentions. To surmount this limitation, future studies might contemplate employing quantitative analytical methods such as mathematical modeling and system dynamics simulations. Such methodologies could facilitate the simulation and prediction of users' information sharing intentions and behaviors across time, thereby offering a deeper understanding of the pathways and patterns influenced by various factors. (please see lines 482-492).

Secondarily, in scrutinizing information sharing behaviors within the social media milieu, this study espoused a relatively macroscopic perspective, viewing social media as a homogenous entity. However, this approach neglected to sufficiently account for the distinctive characteristics of differing social media platforms. Indeed, various social media platforms—each endowed with its own unique user demographics, interaction patterns, and content types—may substantially impact users' information sharing behaviors. Henceforth, future research endeavors should aspire to a more granular examination of the specific features of diverse social media platforms and investigate how these features shape users' preferences and patterns of information sharing. (please see lines 493-501).

8. In “Results of reliability and validity” section, introduce what software was utilized for data analyzing.

Author’s Response: We appreciate the reviewer's attention to the clarity of our methods, particularly in specifying the software used for data analysis.

To address this point, we have revised the "Results of Reliability and Validity" section in our manuscript to include details about the software employed in our analyses. Here is the updated text:

“Due tomeasurement errors, the reliability and validity of the questionnaire are tested before the data analysis, ensuring research outcome credibility. Among them, reliability refers to the degree of reliability of the measurement data, and validity refers to the degree of agreement between the observed variables and the latent variables. In this study, SPSS 24.0 software was used to test the reliability and validity, and the data are shown in Table 2.” (please see lines 276-281).

Thank you once again for your dedication and the time you have invested in our research. We look forward to receiving your feedback and are open to any further suggestions that may help us strengthen our work.
